# A quantitative and non-invasive vibrational method to assess bone fracture healing: a clinical case study

**DOI:** 10.1080/23335432.2021.1874528

**Published:** 2021-02-23

**Authors:** Lorenza Mattei, Miriam Di Fonzo, Stefano Marchetti, Francesca Di Puccio

**Affiliations:** a Department of Civil and Industrial Engineering, University of Pisa, Pisa, Italy; b Department of Translational Research and New Surgical and Medical Technologies, University of Pisa, Pisa, Italy

**Keywords:** Fracture healing, external fixation, bone callus, mechanical vibrations, impact testing, resonant frequencies

## Abstract

Orthopaedics needs a robust diagnostic tool that can help or even replace traditional radiography in bone healing assessment, thus reducing patient exposure to ionizing radiation. We used a vibrational method to assess the healing of a complex fracture treated with external fixation, exploiting a quantitative and non-invasive procedure. Callus stiffening was monitored from the time of surgery until the fixator was removed. Our approach overcomes previous limitations and involves a longer period of healing monitoring (about 9 months), very frequent tests (bi-weekly), and the analysis of a single test configuration. The healing process was monitored by analysing the percentage increments of the squared resonant frequencies (SFIs), related to the stiffness variation and the changes in the frequency response functions. The results were validated by X-rays images, and revealed that the most sensitive parameter to quantify the healing was the SFI of the first resonant frequency which increased by about 20% per month during the formation of the woven callus and up to about 50% at the end of healing completion. This study confirms the potential of the vibrational method as an alternative to radiography in fracture healing assessment.

## 1. Introduction

Although radiography is efficient in assessing fracture healing it is invasive as the patient is exposed to ionizing radiation, and interpretations are subjective. Moreover, X-rays cannot be repeated sufficiently frequently to ensure the best quality patient care. This means that non-union or delayed union of fractures may be detected too late, and may even affect rehabilitation by not detecting the right time for load support and fixator removal. These limitations have encouraged the investigation of non-invasive methods for quantifying fracture healing.

Most approaches in the literature are based on bone stiffness estimation (Morshed 2014; Chen et al. 2015) since it is well known that it increases during the evolution from the immature bone to the cortical bone (Richardson et al. 1994; Byrne et al. 2011). A promising method to indirectly evaluate variations in bone stiffness is based on the analysis of the vibrational response of the fractured bone during healing, which is characterized by an increase in the resonant frequencies (RFs).

This method was originally proposed in the 1990s (Cunningham et al. 1990; Nikiforidis et al. 1990; Tower et al. 1993), and has since been revisited by many authors and applied *in-vitro* to investigate the healing of fractures fixated both externally (Bediz et al. 2010; Ong et al. 2016; Mattei et al. 2017; Di Puccio et al. 2017a; Verdenelli et al. 2018) and internally (e.g. intramedullary nail) (Chiu et al. 2019a, 2019b).

Fixated fractures were focused in this and in our previous studies because they are critical and require an accurate assessment of healing for the best patient care, e.g. to determine the right time to remove the fixator and to promptly identify possible pin infection. Additionally, the fixator itself can be used to perform the measurements by using pins screwed directly into the bone, thus reducing problems related to soft tissue damping (Mattei et al. 2017).

Our previous work has demonstrated the validity of impact testing for assessing the healing of fractures treated with an external fixation, firstly *in-vitro* (Di Puccio et al. 2017b)(Mattei et al. 2017; Di Puccio et al. 2017a, 2017b) and then also *in-vivo* (Mattei et al. 2018, 2019). Encouraging results were obtained in *in-vivo* studies, with a significant increment in the RFs, particularly during the development of the woven callus.

The aim of this work was thus to evaluate the feasibility and reliability of the vibrational method to quantitatively assess the healing of a complex tibial fracture and leg lengthening with an external fixation. The three key new aspects consist in: a longer period of healing monitoring (about 9 months), very frequent tests (about every two weeks), and the analysis of a single test configuration. After the fixator had been removed, the dynamic vibrational response of the leg was also investigated.

## 2. Materials and methods

### 2.1. Case study

The case study concerns a 54-year-old male patient with a polytrauma of the right leg, following a car accident, consisting in a mangled extremity above the ankle, fracture of the distal right tibia, Lisfranc fracture-dislocation, right dislocation of the hip combined with acetabulum fracture, as well as olecranon fracture. The patient underwent damage control surgery (at the beginning the mangled extremity severity score (MESS) was 7, underlining the possible need for amputation) and therefore a subsequent definitive leg fixation was achieved using a hybrid external fixator, TrueLok (Orthofix®). A month later, due to skin and muscle necrosis, the fracture was widely exposed and a negative wound pressure therapy (the V.A.C® therapy, KCI) was applied for 20 days; at the removal, the wound was filled using the artificial skin Integra (Integra Life Sciences Corporation). The external fixator was partially removed after four months (since the day of trauma) and stem cells were injected in the fracture outbreak. At eight months, the external fixator was fully removed. Due to an infected non-union, a Masquelet procedure was performed and the V.A.C® therapy was repeated. In fact, the skin graft failed. At 10 months, the latissimus dorsi muscle flap was positioned on the skin lesion, antibiotic-treated cement was removed, and the non-union was compressed by shortening the leg.

**Figure 1.**
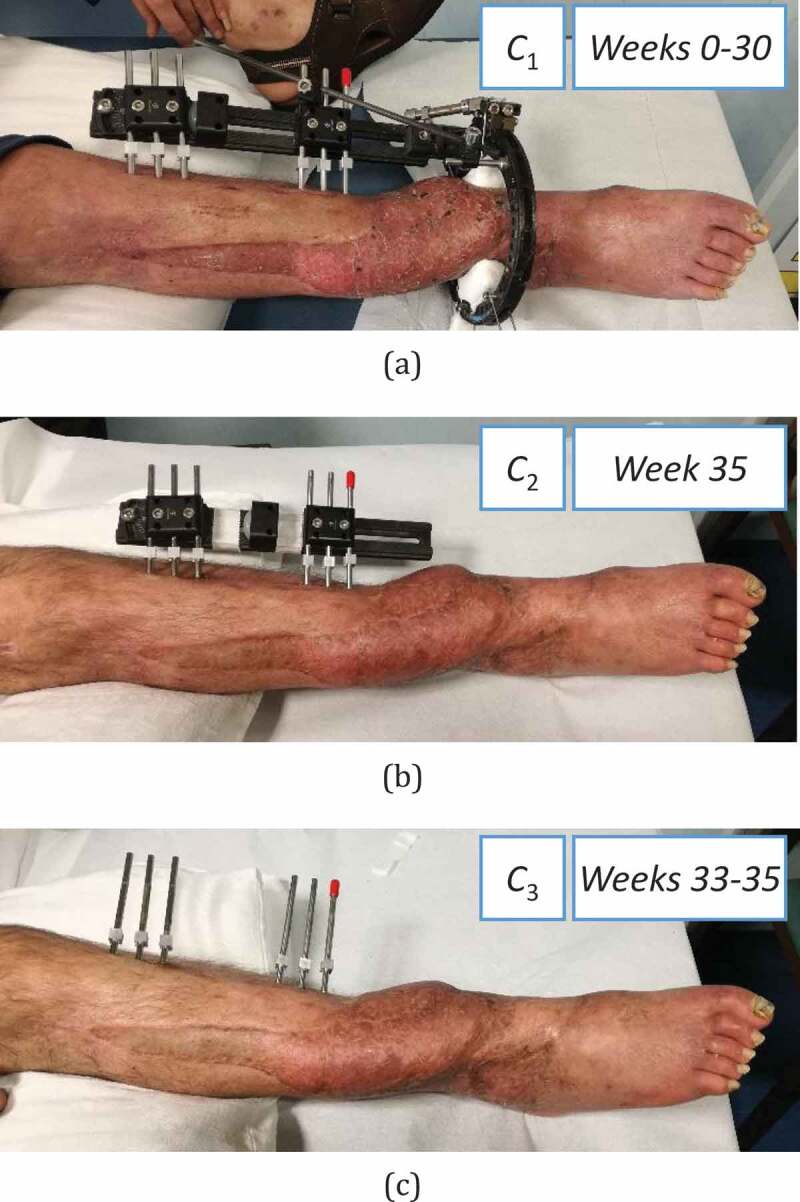
The case study in three different configurations: (a) *C*_1_ at Weeks 0–30, (b) *C*_2_ at Week 33, (c) *C*_3_ at Weeks 33 and 35

The lengthening procedure was carried out using the LRS Orthofix® fixator, which was connected to a distal ring (Figure 1(a)). Due to the complex external fixator frame, it was possible to compress the fracture (non-union) site distally while proximally a progressive lengthening (distraction at the osteotomy level) of 1 mm per day was performed. At 12 months an elongation of 4 cm had been obtained. At 17 months the LRS fixator was free, so that the load was supported completely by the leg. At 20 months the ring was removed (Figure 1(b)), and finally, at about 21 months, the whole fixator was removed (Figure 1(c)).

Informed written consent was obtained from the patient for publication of this report and any accompanying images. The work fully adheres to the Declaration of Helsinki.

### 2.2. Test set-up and procedure

**Figure 2.**
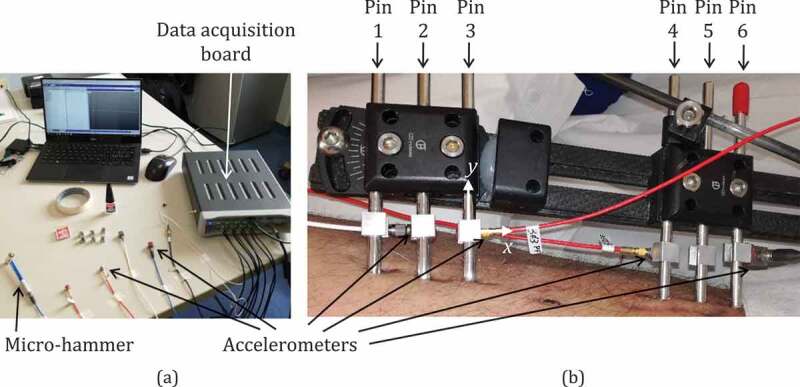
(a) Test instrumentation and (b) accelerometers positioning on fixator pins. As an example, the local reference frame of pin 3 is shown, with the *x* direction almost parallel to the tibia axis, and the *y* direction parallel to the pin axis

**Figure 3.**
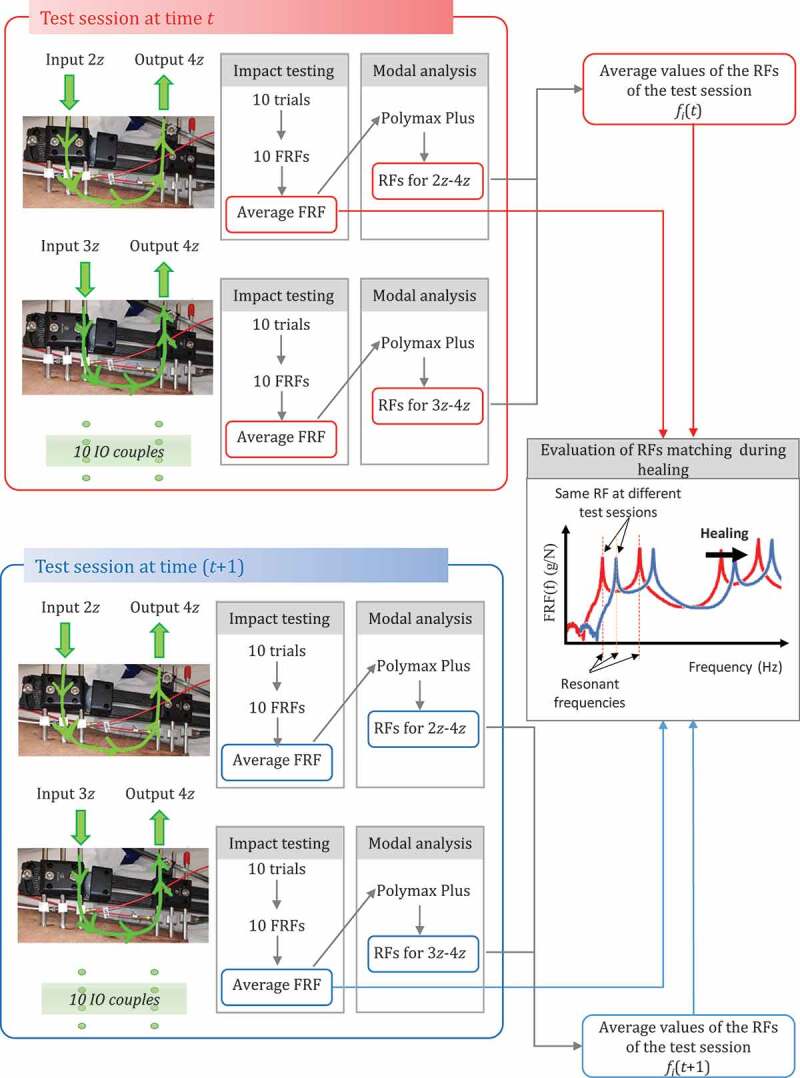
The impact testing procedure and the data processing performed to evaluate the average RFs at each test session and the RFs matching at different test sessions, during healing

The vibrational analysis was based on impact tests whose procedure was defined and applied in our previous studies (Mattei et al. 2017, 2018, 2019; Di Puccio et al. 2017a). The test set-up (Figure 2(a)) consisted in a *Dytran 5800SL* micro-hammer to excite the leg (load level of 0.1 N not perceivable by the patient), and four monoaxial accelerometers (two *3035B Dytran* and two *4507 Brüel & Kjær*) to measure vibrations. The signals were acquired using an LMS Scadas mobile 01 and processed using LMS Test.Lab software. As shown in Figure 2, tests were performed using the Impact Testing package. Signals were acquired in the 0–4096 Hz bandwidth, at a frequency resolution of 2 Hz. To reduce noise artifacts, each measurement was obtained by several impacts, by averaging 10 trials. The data were then processed using Test.Lab’s Modal Analysis package . As in our previous studies (Mattei et al. 2018), data were analyzed in the bandwidth 0–1000 Hz. The frequency response functions (FRFs) were analyzed and the resonant frequencies (RFs) estimated using the PolymaxPlus algorithm, which is particularly efficient for very damped structures (Figure 3).

### 2.3. Test protocol

Osteogenesis and callus stiffening were monitored from the end of the lengthening procedure (12 months after the car crash), i.e. test time zero until the fixator was removed, for 35 weeks. Sixteen test sessions were performed, about every two weeks, for a total of 170 impact tests.

Tests were performed with the patient lying down, with the leg placed on the examination table. Three configurations were examined, shown in Figure 1: *C*_1_, characterized by the leg treated with the LRS Orthofix® fixator, was the reference one for healing monitoring in 14 test sessions throughout Weeks 0–30 (Figure 1(a)); *C*_2,_ characterized by a simplified fixator configuration, without the ring, and evaluated at Week 35 (Figure 1(b)); *C*_3_, corresponding to the healed leg after fixator removal, with only pins, and tested twice, at Weeks 30 and 35 (Figure 1(c)). *C*_3_ measurements were used to evaluate the leg vibrational response and its resonant frequencies, which are rarely reported in the literature (Mattei et al. 2018, 2019), as discussed in (Mattei et al. 2019).

The pins screwed in the bone were used to transfer both input and output signals. Cubic supports glued on pins were used both to excite the leg and apply accelerometers, as shown in Figure 2(b). During each test session, 10 couples of input-output (IO) were considered, as described in Table 1. The input and output directions were parallel to the *x* and *z* axis of pin local frames with the *x* direction almost parallel to the tibia shaft and the *y* direction parallel to pin axis, as in the example in Figure 2(b).

**Table 1.** Input and output couples: location on pin and direction, defined according to the local reference frames of the pin (see Figure 1(b))

IO couple	Input	Dir.	Output	Dir.
1x-4z	Pin 1	-*x*	Pin 4	+*z*
1x-6x	Pin 1	-*x*	Pin 6	-*x*
2z-4z	Pin 2	+*z*	Pin 4	+*z*
2z-6x	Pin 2	+*z*	Pin 6	-*x*
3z-4z	Pin 3	+*z*	Pin 4	+*z*
3z-6x	Pin 3	+*z*	Pin 6	-*x*
4z-2z	Pin 4	+*z*	Pin 3	+*z*
4z-3z	Pin 4	+*z*	Pin 3	+*z*
5z-2z	Pin 5	+*z*	Pin 2	+*z*
5z-3z	Pin 5	+*z*	Pin 3	+*z*

Hereafter, IO couples are indicated as, for instance, 3*z*-4*z*, where the numbers indicate the input/output pin (pin 3 as input and pin 4 as output), and the letters indicate the excitation/measurement direction (*z* direction). These IO couples were selected based both on the results of our previous *in-vitro* and *in-vivo* investigations, and on the fixator configuration. Three key factors influenced this selection:

i) the most significant measurements were expected when testing input and output pins over the fracture site, from signals in a direction normal to the bone axis (Mattei et al. 2017, 2018, 2019; Di Puccio et al. 2017a);

ii) some directions were not feasible;

iii) tests using IO couples with the same input and close outputs (e.g. 4z-3z and 4z-2z), and vice versa, provided almost equal measurements (Mattei et al. 2018, 2019).

### 2.4. Data processing, presentation and quality

Since all the IO couples described in Table 1 were tested for each experimental session, a large amount of data was collected and processed as described in Figure 3. In each test session, 10 trials were performed for each IO couple, 10 FRFs were obtained and averaged in order to provide a single FRF (per IO couple per session) from which RFs were estimated. As expected, many frequencies (denoted with *f_i_*) were obtained in a 0–1000 Hz range, and differed from session to session. Thus, a very crucial point was matching the RFs of consecutive sessions, which is fundamental for evaluating healing. This matching process was based on a comparison of the shape of the average FRF for single couples (i.e. matching of FRF peaks), Figure 3.

**Figure 4.**
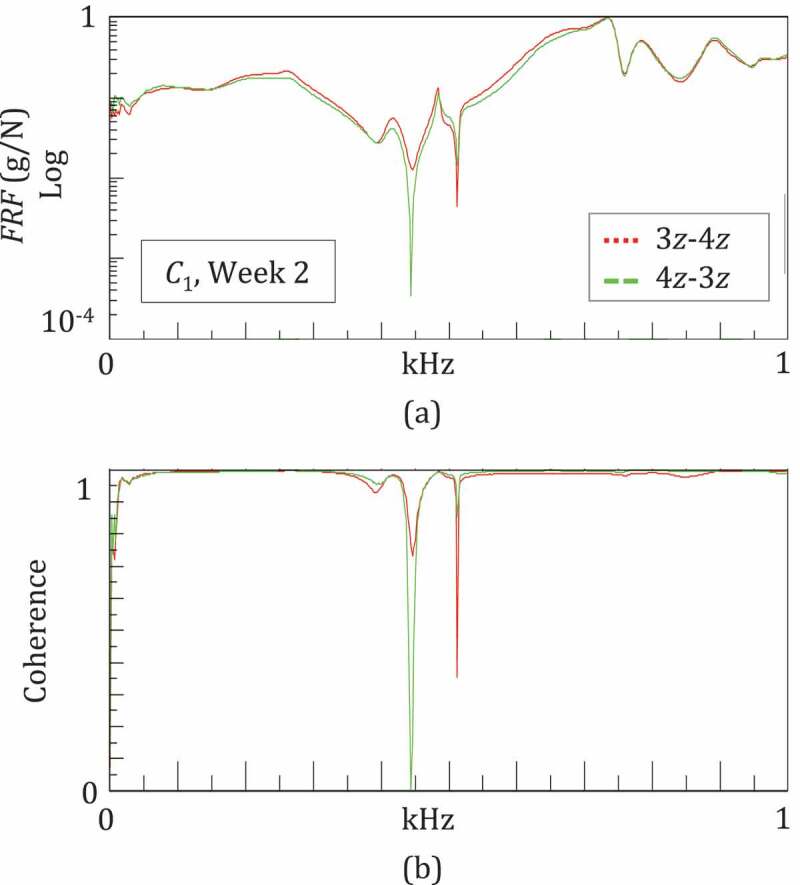
Example of the system linearity and measurement quality given by a good reciprocity and a high coherence

All the operations outlined above were based on the hypotheses of system linearity and good quality data. These two properties were preliminarily checked for all sessions/configurations by evaluating the system reciprocity and the coherence, respectively (Ewins 2000). The reciprocity is verified when the same FRFs are obtained by exchanging IO points and directions. The coherence function is obtained by computing the input and output power spectra and their cross-power spectrum, and can vary in the range 0–1, where 0 implies a non-linear relation between input and output, whilst 1 means a perfect linear relationship (Ewins 2000). Good quality measurements are proven by a coherence close to 1 (>0.9). As an example, Figure 4 describes the check process for the second test session in *C*_1_: the FRFs for 3z-4z and 4z-3z are almost overlapped (Figure 4(a)), and the coherence functions, close to 1 (Figure 4(b)).

The goodness of the data was also highlighted by low differences (<5 Hz) among *f_i_* values estimated from different IO couples in a given test session. In fact, the average deviations were about 2.5 Hz, comparable to the frequency acquisition resolution of 2 Hz.

The next step consisted in introducing the percentage squared frequency increments (SFIs), since these are proportionally related to the stiffness variation

(1) $$SFI_{i}\left(wk\right)=\frac{f_{i}{\left(wk\right)}^{2}-f_{i}{\left(wk_{0}\right)}^{2}}{f_{i}{\left(wk_{0}\right)}^{2}}100$$

The SFIs of a given RF were computed with respect to the RF value at its first appearance at *wk*_0_ (e.g. *wk*_0_ corresponds to Week 0 for *f*_1_, *f*_2_, *f*_3_ and to Week 21 for *f*_12_). Finally, in order to condensate the results, the trend of the sum of selected SFIs was considered during healing.

## 3. Results

### 3.1. Fracture healing monitoring

The callus distraction healing process was assessed using two approaches: i) a quantitative comparison of the RFs and SFIs, and ii) a qualitative comparison of the FRF trends during healing. Table 2 reports values of the RFs obtained from the test campaign; each *f_i_* column corresponds to a vibrational mode.

**Table 2.** Average values of the RFs for configurations *C*_1-3_, over a period of 35 weeks, for a total of 16 test sessions. Note that each RF *f_i_*/column corresponds to a vibrational mode

Test sess.	Time (Weeks)	Conf.	*f*_1_	*f*_2_	*f*_3_	*f*_4_	*f*_5_	*f*_6_	*f*_7_	*f*_8_	*f*_9_	*f*_10_	*f*_11_	*f*_12_	*f*_13_
1	0		83	259	409	512	620	694	713		764	877	894		920
2	2		83	263	416	514	620	705	718	730	764	875	890		923
3	4		92	264	411	513	620	699	718	730	774	875	903		926
4	7		95	260	413	511	622	707	723		768	874	917		932
5	9		94	263	410	511	624		721		765	869	914		930
6	10		95	260	412	511		712	723	736	765	871	921		930
7	13	*C*_1_	97	260	409	510		716	724	739		879			933
8	15		97	264	425	514	622	717	727	738	772	876			934
9	19		97	264	413	514	625	713	724	739	773				
10	21		96	268	412	514			729	751	776	882		901	938
11	23		98	264	414	513	624	720	738	759	773	882		909	952
12	25		100	267	421	514	623	723	746		778	894		915	958
13	27		98	266	422	514	628	730	746	756	785	904		922	957
14	30		101	265	424	516	629	736	753	760	794	908		922	960
15	33	*C*_2_	66	139	199	296	389	607	683	753	865				
		*C*_3_	60	121	190	268	355	498	714	856	899	962			
16	35	*C*_3_	65	120	188	266	363	502	711	853	909	959			

The configuration *C*_1_ was characterized by 13 RFs: four in 0–0.55 kHz, and nine in 0.55–1 kHz. Not all the RFs were visible in each test session: we were only able to monitor RFs *f*_1-4_ and *f*_7_ throughout the entire healing period. Table 2 shows that the healing caused an overall increase in all the RFs. The highest increase was for the first RF *f*_1_, which moved from 83 Hz to 101 Hz in 30 weeks. This increase occurred in two main steps: from Weeks 2 to 7 and from Weeks 21 to 30.

**Figure 5.**
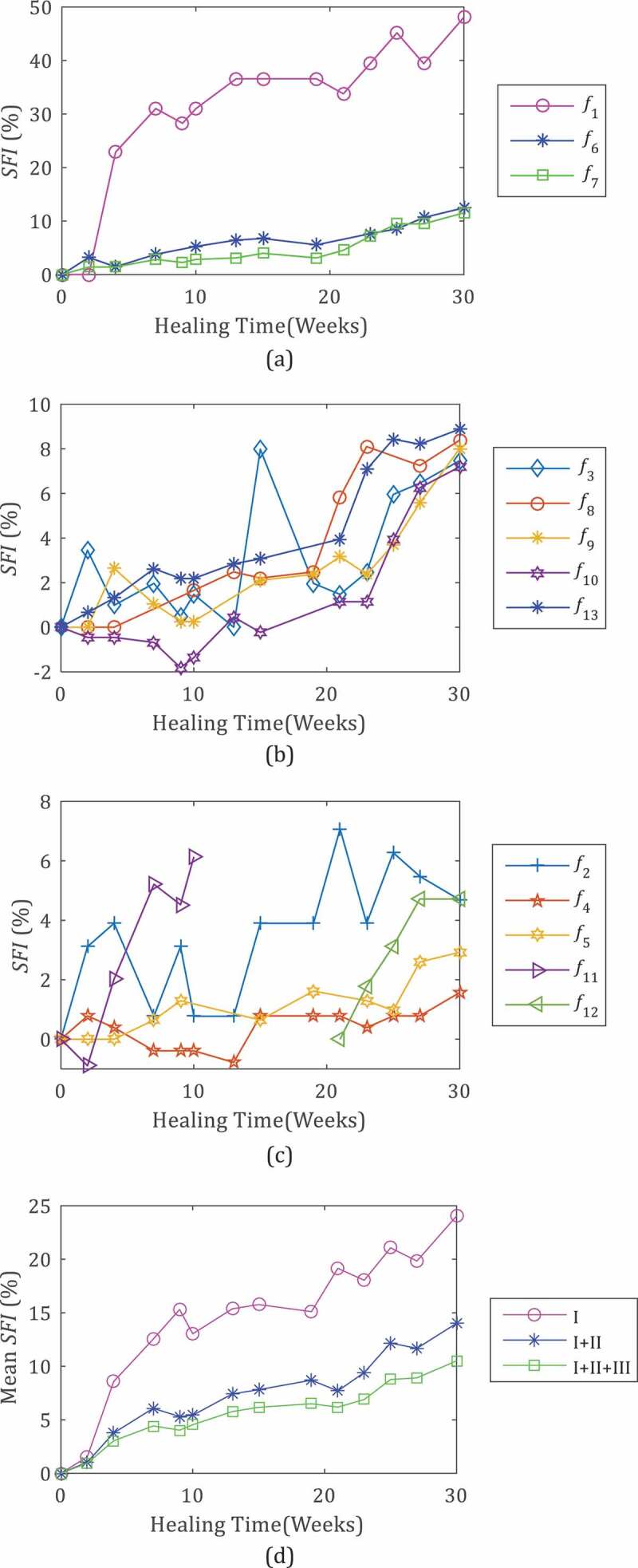
Percentage squared frequency increment (*SFI*) of the leg RFs during the healing (*C*_1_, Weeks 0–30) (a–c), and mean SFI evaluated considering only frequencies in Group I (*f*_1_, *f*_6_, *f*_7_) (I), Group I and Group II (*f*_3_, *f*_8_, *f*_9_, *f*_10_, *f*_13_) (I+ II) and all SFIs (I+ II+III) (d)

The SFI curves of the RFs are shown in Figure 5(a–c). They are in three groups:

a) Group I – frequencies *f*_1_, *f*_6_, *f*_7_ that experienced the greatest variation during healing with final SFIs higher than 10%, (48%, 11.5% and 12.5%, respectively) (Figure 5(a));

b) Group II – *f*_3_, *f*_8_, *f*_9_, *f*_10_, *f*_13_ with a medium increment with a final SFI in the range 5–10%, more precisely 7.2–8.9% (Figure 5(b));

c) Group III – *f*_2_, *f*_4_, *f*_5_, *f*_11_, *f*_12_ with small variations, SFIs lower than 5%, in the range 1.6% and 4.7%.

The callus stiffening mostly affected the first frequency *f*_1_ which showed an *SFI*_1_ of about 50% when fully healed, increasing rapidly at the beginning (up to 31% in 7 weeks, at about 21.2%/month) and then more slowly (at 2.8% per month).

Figure 5 highlights the different trends of the RFs; for example, *SFI*_7_, *SFI*_9_, *SFI*_10_ and *SFI*_13_ increased more quickly in the last weeks than in the first weeks, whereas *SFI*_5_ did the opposite. These different trends of the *SFI*s can be explained considering that fracture healing is a very complex process affected not only by callus stiffening, but also by the evolution of the initial hematoma, leg inertia, muscle tone and so on. Consequently we did not base healing monitoring on a single *SFI* but also on the mean value of some or all *SFI_s_* at a given week. In particular, Figure 5(d) compares the trends of the mean SFI evaluated considering only frequencies in Group I, Group I and II, and all groups. In all the three cases, the averaging smooths the local variations in *SFIs* and highlights the trend observed for *SFI*_1_ characterized by two steps: an initial rapid increase in Weeks 0–7, followed by a slower increase in Weeks 7–30.

**Figure 6.**
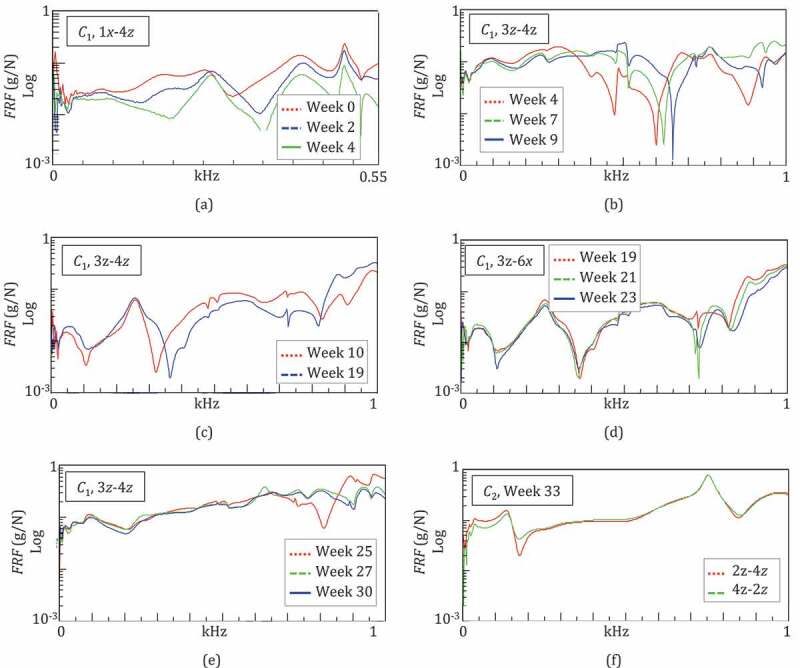
Monitoring of the fracture healing by means of the temporal evolution of the leg FRFs in configuration *C*_1_ (a–e). Comparison of the FRFs in configuration *C*_2_ exchanging IO: example of good reciprocity (f)

The temporal evolution of the FRFs during healing is plotted in Figure 6 for the IO couples that most clearly revealed the healing in specific periods. The frequency responses of the leg during the first month were similar at the low frequencies (under 550 Hz), but varied significantly at the high frequencies (over 550 Hz), where the comparison of FRFs was not significant. Figure 6(a) shows the evolution of FRFs obtained for 1x-4z at Weeks 0–4, under 550 Hz: the curves had similar trends with characteristic peaks at the RFs and, from Week 0 to Week 4, shifting towards the high frequencies. The RF values in Table 2 indicate that the shift was more marked for the first two peaks/RFs, between Weeks 2 and 4. This suggests that the callus began stiffening approximately two weeks after the end of the lengthening procedure.

During Weeks 4–9, the frequency responses of the system were compared in the bandwidth of 0–1 kHz, as shown in Figure 6(b) for IO 3z-4z. The FRFs were similar and almost overlapped at the low frequencies, under 350 Hz, whilst at high frequencies, over 600 Hz, they appeared to be shifted, demonstrating that the healing was progressing slowly.

During Weeks 10–19, the FRFs were comparable over the whole bandwidth of 0–1 kHz, with a weak shift of the FRF at Week 19 with respect to Week 10, as shown in Figure 6(c) for IO 3z-6x. This was in agreement with the weak increase in all the RFs (Table 2) and demonstrated that the healing status was advanced.

In fact in weeks 25–30, the frequency responses were very similar as indicated in Figure 6(e) for 3z-4z. Only a change above 700 Hz was observed in FRF trends from Week 25 to Week 27. Most of the FRF peaks slightly shifted towards high frequencies, since all the RFs increased, with the exception of *f*_1_ and *f*_2_ which remained almost constant (Table 2). This confirmed the completion of the healing process.

Configuration *C*_2_, where the ring was removed and the fractured bone was almost healed, was characterized by nine RFs. An example of an FRF obtained for IO 2z-4z in *C*_2_ is given in Figure 6(f): the good reciprocity of the system was verified by almost identical FRFs obtained when exchanging IO. A comparison with *C*_1_ was not possible since the FRFs corresponded to different structures. However, the frequency response of the leg in *C*_2_ appeared to be simpler than that of *C*_1_, with a lower number of peaks, which were even more pronounced, as a consequence of a simplified fixator structure.

### 3.2. Detection of infection at pin

**Figure 7.**
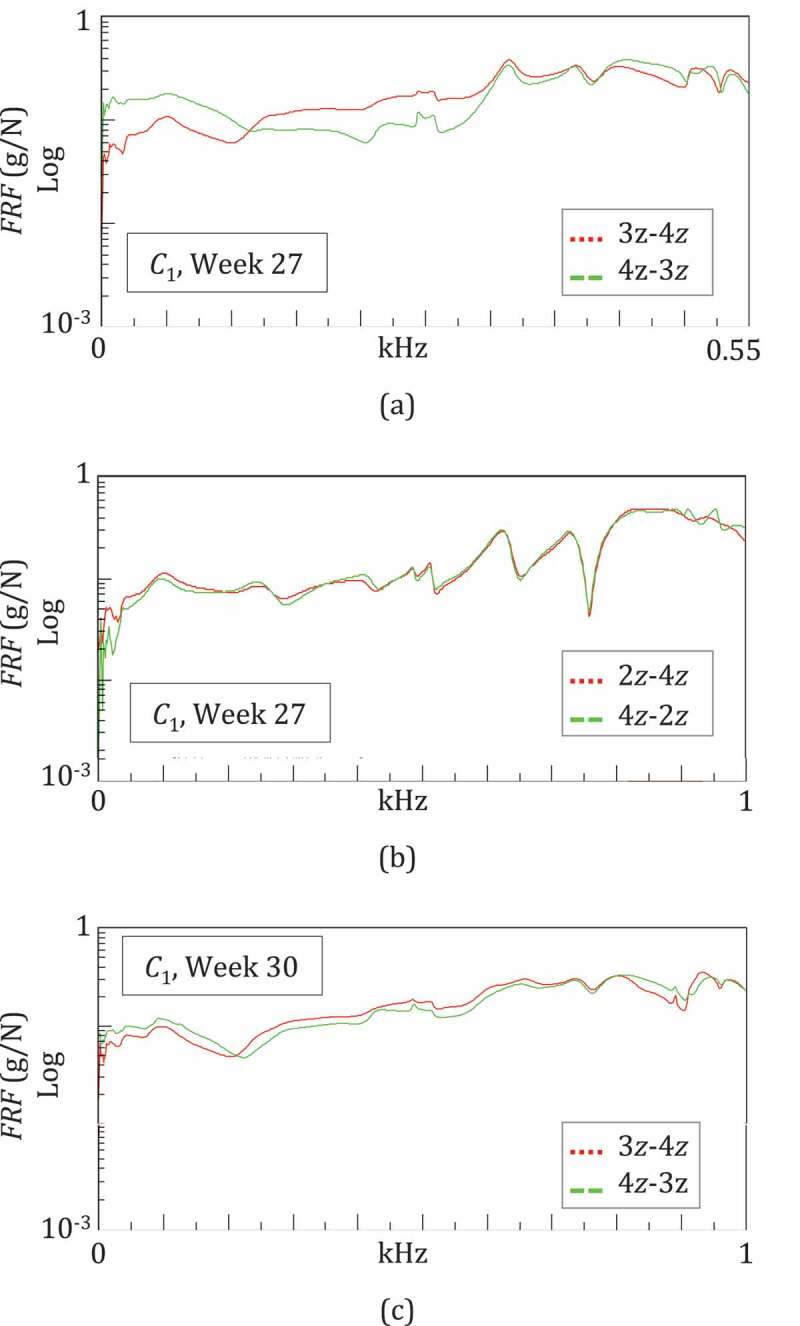
Detection of pin infection: non verified reciprocity for 3z-4z and the verified reciprocity for 2z-4z suggests the infection of pin 3 at Week 27. The improved reciprocity for 3z-4z at Week 30 suggests a reduction in pin infection

At Week 27, the preliminary reciprocity tests using both pins 1 and 3 as input, and pin 4 as output failed. Two different FRFs, although with similar trends and correspondent peak locations, were obtained by exchanging the IO, as shown in Figure 7(a). On the other hand, reciprocity was assessed using other IO couples, i.e. pin 2 and again pin 4 (Figure 7(b)).

**Figure 8.**
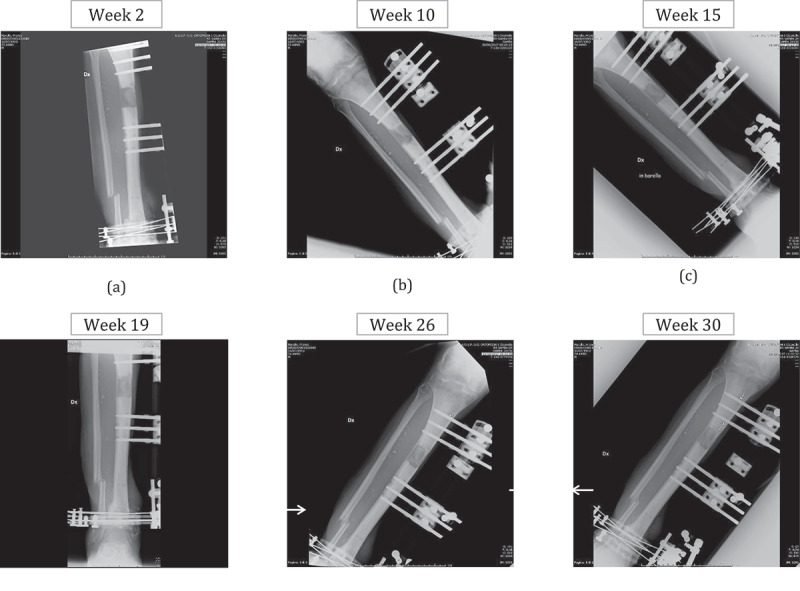
X-rays during the fracture healing

At Week 30, the reciprocity tests were repeated: there was an improvement for IO 3z-4z (Figure 7(c)), whilst the test failed again for IO 1x-4z. These observations were confirmed in the following sessions, in *C*_2_ and *C*_3_. These results suggested that something happened to pins 1 and 3 at around Week 27. The radiographical findings (see 3.2.3), at Week 26 revealed infections at pins 1 and 3, with some loosening (see Figure 8). The subsequent X-rays at Week 30 showed that the infection of pin 3 had regressed while reciprocity improved (Figure 8).

These observations support the hypothesis that pin infection can be detected by a missing reciprocity in impact tests. On the other hand, the RF values estimated using an infected pin as I/O were equal to those estimated using healthy pins.

### 3.3. Results validation by means of X-rays

The results were validated by X-ray images taken every 4–6 weeks, against the bi-weekly occurrence of impact tests (6 X-ray controls vs 16 vibrational controls in 30 weeks). The most significant radiographic images are shown in Figure 8. In order to evaluate the osteogenesis process, both the shape and type of the callus were considered (Li et al. 2006). At Week 2, a soft woven callus was visible at the fracture site (osteogenesis callus shape 2, type 3), revealing that the healing process began as soon as it had been triggered (Figure 8(a), osteogenesis callus shape 2, type 7), as demonstrated by impact tests. At Week 10, the woven callus appeared together with the soft callus (Figure 8(b)) confirming the increase in callus stiffness predicted by the vibrational tests, i.e. an increment in the RF values, particularly of the first one *f*_1_. X-rays taken at Weeks 15 and 19 showed that the woven callus gradually evolved into a hard callus Figure 8(c–d) (osteogenesis callus shape 2, type 7–10). In fact, the leg frequency responses in the following weeks did not vary considerably.

X-rays at Week 26, showed the consolidation (osteogenesis callus shape 2, type 10) of the fracture site, although there were some areas of bone rarefaction at pins 1 and 3 (indicated by arrows in Figure 8(d)), reflecting the initial alisteresis and loss of tightness of the screws into the bone. Finally, the X-ray at Week 30 (before the ring removal) confirmed both the healing and the initial alisteresis, although the latter was lower in pin 3 than in the previous month (Figure 8(f)).

### 3.4. Leg vibrational response

**Figure 9.**
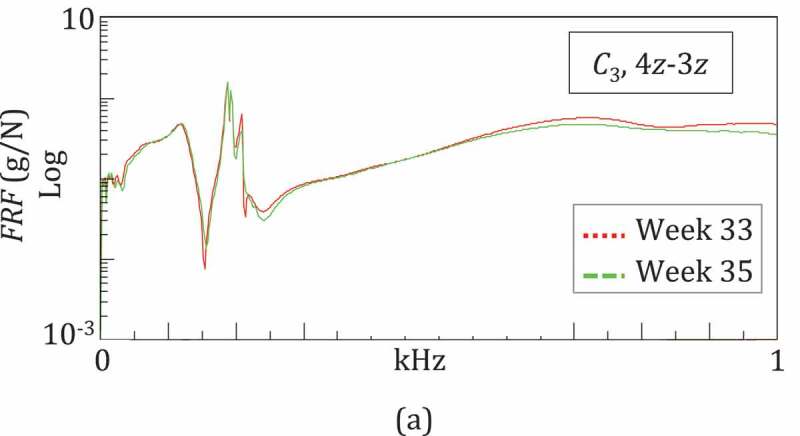
Vibratory response of the healed leg after fixator removal: very similar trends of FRFs at Weeks 33 and 35 confirmed the completion of the healing process

The frequency response of the leg was also evaluated after the fixator had been removed, at Weeks 33 and 35 (configuration *C*_3_). The leg was characterized by 10 RFs in 0–1 kHz, whose values at Week 33 were 60, 121, 190, 268, 355, 498, 714, 856, 899 and 962 Hz. After two weeks, only *f*_1_ had a small increment up to 65 Hz. The frequency responses in this period were almost unchanged, as demonstrated by the comparison of the FRFs obtained for IO 4z-3z in Figure 9. This confirmed the completion of the healing process.

## 4. Discussion

### 4.1. Comparison with the literature

The vibrational method has only rarely been used to monitor *in-vivo* fracture healing (Cunningham et al. 1990; Nikiforidis et al. 1990; Tower et al. 1993; Mattei et al. 2018, 2019), and in fact (Mattei et al. 2018, 2019) were conducted by our team.

The present study confirms previous results in two key areas:

i) the vibrational response changes mainly during the transformation of the soft callus into the woven callus (Weeks 0–7) but it does not vary significantly in the subsequent healing phases.

ii) the first RF is the most sensitive to callus stiffening.

On the other hand, in this study the healing process and thus the bone stiffening was slower with respect to previous studies (Mattei et al. 2018, 2019) and the linear rate of *SFI*_1_ during the woven callus development was almost halved, i.e. 5% per week vs 7–10% per week.

The dynamic response after the fixator had been removed was also investigated in (Mattei et al. 2018, 2019). However, a comparison can only be qualitative, considering the many differences between the case studies such as the fracture site (femur and leg), the patient’s age and muscle tone. The FRFs obtained in the present study and in (Mattei et al. 2018, 2019) were all characterized by clear and high peaks at the RFs at the low frequencies (0–400 Hz), and very weak peaks at the high frequencies (400–1000 Hz).

### 4.2. Limitations for transfer to clinical practice

A critical point of data processing is matching the RFs that derive from different test sessions, which requires a skilled operator. It could be improved and performed automatically by means of modal forms reconstruction which will be the next step in our research.

Another fundamental issue in applying the method is to define a reliable indicator together with its threshold values to identify non-unions or delayed unions from a positive healing progression. In this study, we have proposed the squared frequency increment of the first RF or of a combination of RFs. These indicators should be tested on a large scale in order to be reliable and maybe to replace X-rays.

The clinical application of the approach requires other simplifications in order to be performed by non-expert operators. For example, an automatic modal hammer could be considered for vibrational excitation so that impact testing could be performed by orthopaedic surgeons or even by patients. In this way, impact testing, which only requires a portable setup, could be performed both at the hospital and at home, thus in places where radiology laboratories are not available, such as in underdeveloped countries and for the military.

## 5. Conclusions

We have demonstrated the efficacy of vibrational tests to quantitatively assess the healing of a very complex tibia treatment. The results were validated by means of X-ray images. Although the focus was on a single case study, the particularly complex clinical history of the patient corroborates the applicability of the method even in a difficult scenario.

Our vibrational method managed to detect the various healing stages, from the soft callus to the hard callus formation. We exploited a squared frequency increment (SFI) to monitor healing. The SFI of the first resonant frequency was the most sensitive quantity for healing assessment, with an increase of up to 48%. Several SIFs can be used to ensure a more ‘stable’ indicator that is not affected by specific oscillations of a single quantity. We hypothesise that the reciprocity test can be a simple and fast method for detecting pin infection, which is a recurrent and serious complication of fracture fixation.

In order to be exploited in clinical practice and perhaps replace X-rays, two key future steps are needed: i) data should be collected from many different cases so to perform a statistical analysis and to validate indicators and their threshold values; ii) the process needs to be automated and specific hardware and software are required in order for the method to be used by non-expert operators.
